# Supplementary material for: Are carbon emissions trading and green financial instruments synergistic? -Comprehensive quantitative research based on content analysis
Source: PLoS One. 2024 Mar 7;19(3):e0298601. doi: 10.1371/journal.pone.0298601 (PMC10919601; doi:10.1371/journal.pone.0298601)
Supplement: S1 Data — (ZIP) [file pone.0298601.s001.zip › Data/地级市Z_F工作报告.zip!/╜¡╬≈/─╧▓2╩╨/└┤╫╘╣1⁄2╓┌║┼í╛┬φ┐╦╩2╛▌═°í┐_2012.docx]

各位代表：

　　现在，我代表南昌市人民政府，向大会作政府工作报告，请予审议，并请各位市政协委员和列席会议的同志提出意见。

　　一、2011年工作回顾

　　2011年是“十二五”规划的开局之年。面对严峻复杂的国内外发展形势，在省委、省政府和市委的坚强领导下，全市上下坚持以科学发展观为统领，以“创建办会”为契机，按照“六个率先”的工作要求，攻坚克难，务实奋进，全力推进经济社会平稳较快发展，完成了市十三届人大六次会议确定的主要目标任务。全年完成财政总收入411.19亿元，增长27.6%；地方财政一般预算收入187.03亿元，增长27.7%；预计地区生产总值2560亿元，增长13%；500万元以上固定资产投资2001亿元，增长26%；规模以上工业增加值760亿元，增长18%；按在地口径社会消费品零售总额910亿元，增长19%；实际利用外资22.61亿美元，增长12%；实际利用内资585.64亿元，增长15%；进出口总额76亿美元，增长52%；城镇居民人均可支配收入20743元，增长13.5%；农民年人均纯收入8130元，增长13%；万元GDP能耗下降3%。受宏观环境影响，居民消费价格总水平涨幅控制在5.0%，稍高于4%左右的年度目标。

　　（一）创建办会成果丰硕，城市形象彰显新魅力

　　2011年是我市的“创建办会”年。按照“创建为民、创建惠民、创建便民”的要求，认真开展了全国文明城市、全国双拥模范城市等创建工作。成功举办了赣台会、泛珠会、世界低碳与生态经济大会等大型活动。特别是“七城会”作为建国以来南昌乃至江西举办的规模最大、层次最高、影响最广的体育盛会，高标准完成了场馆建设、大型活动、赛事组织、安全保卫、新闻宣传等各项工作，为全国人民呈现了一届“精彩难忘、绿色生态、欢乐祥和、节俭高效”的城运盛会，展现了“红色英雄城、绿色七城会”的靓丽风采，实现了“出形象、上水平、聚人心”的预期目标，得到了党中央、国务院和省委、省政府以及参赛人员和社会各界的充分肯定。

　　（二）经济运行总体稳健，三大产业迈上新台阶

　　工业经济平稳增长。148个超亿元项目进展顺利，投资百亿元的低碳产业园和区域总部基地项目成功签约；列入省战略性新兴产业重大项目的格特拉克DCT等11个项目集中开工；江铃30万辆整车、南昌航空工业城等项目稳步推进；双汇肉制品加工、新华印刷等项目顺利竣工；赛维LDK1000兆瓦模组件、江铜10万吨铜板带（一期）等项目全面投产。全市规模以上工业企业增至938户，省级以上工业园区实现主营业务收入2180亿元，增长25%。

　　现代服务业加快发展。南昌华南城、万达广场、家乐福等56个重大服务业项目开工开业，华润集团成功并购洪客隆；我市被列为全国再生资源回收利用、肉菜追溯和农产品现代流通三大体系试点城市；顺利完成16家集贸市场升级改造，建设3个“万村千乡”配送中心和300家农家店，家电以旧换新销售额占全省50%左右。北京银行南昌分行正式开业，新增村镇银行2家；小额贷款公司实现县区全覆盖；恒大高新成功上市，上市公司再融资28.6亿元。接待国内外游客2008万余人次，实现旅游总收入135.1亿元。成功举办中国绿博会等130余项展会，连续5年荣获“中国十佳会展城市”称号。

　　农业经济稳中有升。落实强农惠农富农政策，发放各类农业补贴6.22亿元。预计农业总产值232.7亿元，增长6%；粮食总产量241.2万吨，蔬菜总产量156万吨，肉类总产量35.57万吨，水产品起水量35.8万吨。市级以上农业产业化龙头企业达到321家，新增无公害农产品基地25个。启动12个大型农业综合开发项目，实施造地增粮富民工程30.74万亩，建设现代农业示范区20.7万亩。推进乌沙河综合整治和修河等5条中小河流治理，完成59座小型水库除险加固。湖坊镇财政总收入在全省率先突破10亿元。

　　（三）改革开放持续深入，体制机制再添新活力

　　重点领域改革成效明显。顺利完成364户非工口七大系统国有企业改革。南昌县、进贤县、东湖区、青山湖区被批准为省综合配套改革试点县区，南昌县被列为国家城乡统筹一体化试点县。市与区共同发展、财源共生、利益共享的收入增长机制和财力分配机制更加健全。户籍制度改革深入实施，企业统计、事业单位人事和绩效工资等改革有序推进。土地统一上网挂牌出让、争取促进产业发展配套和城市建设融资政策等方面走在全省前列。医药卫生体制改革深入推进，政府举办的基层医疗机构全部实施国家基本药物制度，公立医疗机构药品省级网上集中招标采购政策顺利实施。非公经济门槛进一步降低、总量进一步壮大，预计全年完成增加值1470亿元，增长13.1%。

　　对内对外开放水平提升。组织参与香港招商引资活动周、第六届中部博览会等重大招商活动32场。新批外资企业184家。引进投资超千万美元外资项目（含增资项目）73个，超亿元内资项目58个。“服务外包示范城市”实现保牌进位，接包合同执行金额13.1亿美元。全球首个开源技术公有云——中国鄱湖云计算中心正式运营。南昌出口加工区、保税物流中心和海铁联运平稳运行。南昌与奥地利低碳合作上升到国家层面务实推进。我市连续8年被评为台商投资“极力推荐城市”。

　　（四）城镇化战略深入实施，城乡面貌有了新改观

　　城乡规划加快完善。《南昌市近期建设规划（2011—2015）》、《南昌市干线路网规划》、《城市公共交通系统规划》等规划编制完成。四县总体规划及省级示范镇总体规划编制工作加快推进。《重点地段规划控制导则》深入实施，违章建筑查处力度加大，规划的权威性进一步增强。

　　城乡建设力度加大。实施新型城镇化项目273项，完成城市建设总投资420亿元，增长27.6%。全年新增中心城区建成区面积6.9平方公里，全市城镇化率提高1.3个百分点，达到67%。交通运输方面，昌北机场新航站楼投入使用，滨江路改线、艾溪湖大桥、青山湖中北大道、沿江中北大道连通工程、德昌高速、昌铜高速等重点工程全面竣工，轨道交通1号线一期工程、南昌西客站建设加快推进，昌南大道东延、生米大桥西延、沿江中南大道等3条快速路开工建设。社会事业方面，南昌国体中心、湾里射击中心等体育场馆及配套路网建设全面完成。公用市政方面，新建及改扩建自来水厂3家，置换天然气用户15万户。城市改造拓展方面，朝阳洲片区改造加速实施，“五大组团”建设加快推进。村镇建设方面，新增农村公路388.6公里，实施渡改桥29座，完成713个省批新农村村点整治，30个重点示范村和 8个重点示范镇建设加快推进，2483个村点农村清洁工程全面铺开。

　　城市管理科学有效。制定了《南昌市城市管理标准》（试行）。投资30亿元实施“2610工程”，对城区175条主次道路和街巷支路、48座桥涵、6大水域、88个社区综合环境进行了改造提升，市容市貌焕然一新，各方宾客眼前一亮。实施公交优先战略，设置“一纵一横”公交专用道，新增10条公交线路，开通了市区至安义县公交线路，增加投放出租汽车342辆，完成南昌长途汽车总站搬迁，启动公路主枢纽“四站一中心”建设。“啄木鸟”行动有效开展，处理各类城市管理问题1.98万件。认真开展“我奉献我快乐”志愿服务活动。强力开展河道采砂专项整治，非法采砂现象得到有效遏制。

　　城乡环境持续优化。深入推进“森林城乡、花园南昌”建设，完成造林26.3万亩，森林覆盖率21.96%、城市绿化率42.8%、人均公园绿地面积9.02平方米。首批命名市级生态村156个。喜迎“七城会”摆放鲜花3000万余盆。开展幸福渠水系综合治理等环保专项行动，关停取缔185家违法排污的企业及养殖户，完成四大主要污染物指标年度减排任务，集中式饮用水源地水质达标率100%，空气质量优良率95.75%。

　　（五）民生事业全面推进，公共服务实现新提升

　　民生状况持续改善。共投入200亿元实施160项民生项目，其中政策性项目全面超额完成，建设类项目完成年度目标任务。城乡就业进一步扩大，新增城镇就业8.12万人，新增转移农村劳动力4.1万人，零就业家庭就业安置率100%，发放小额贷款4.32亿元。社会保障体系更加完善，城镇基本医疗保险和新农保基本实现全覆盖，社会保险基金征缴总量42.57亿元，列全省第一。社会救助水平得到提升，启动了社会救助和保障标准与物价上涨挂钩联动机制，超额完成城市低保、农村低保、农村五保、临时救助、精减退职老职工救济、农村医疗救助、城市医疗救助等七项民生考核任务，全年保障城乡低保对象11.1万户、22.8万人，发放五保供养资金4412.7万元。教育助学深入推进，实现免费义务教育、济困助学及进城务工人员子女免费接受义务教育“三个全覆盖”。安居工程深入实施，11371套保障性住房建设按计划推进，发放经济适用住房货币补贴4790户、23966万元和廉租住房家庭补贴9908户、3453万元，棚户区拆迁改造加快推进。扶贫开发进展顺利，全面实施100个贫困村整村推进扶贫，10个扶贫主导产业示范点进一步巩固。农村人口饮水安全、“光明·微笑”行动、儿童“两病”免费救治、计划生育奖励扶助、孤残儿童保障等各类民生工程顺利推进。

　　社会事业协调发展。南昌十九中、南昌一中等新校区加快建设，新改建农村校舍206万平方米，消除D类危房22万平方米。出台《中长期人才发展规划纲要》和“人才管理改革试验区”建设十项举措，引进355名高层次人才。实施科技创新“六个一”工程，已培育硅基半导体照明工程技术研究中心等3家国家级工程技术研究中心和重点实验室；“十城千辆”和“十城万盏”工程扎实推进，顺利通过国家知识产权质押融资试点工作验收。全部社区卫生服务机构和村卫生所达到建设标准，为19万名市民免费提供健康体检。新四军军部旧址陈列馆和八大山人纪念馆改扩建顺利竣工并免费开放，成功举办第四届国际军乐节、首届南昌·中国雕塑艺术节，我市被评为全国首批集邮文化先进市。“七城会”上南昌代表团夺得18枚金牌，创历史记录；“体育进社区”活动加快推进。人口出生性别比综合治理工作成效明显，全市低生育水平保持稳定。

　　（六）依法治市成效明显，发展环境得到新优化

　　政府自身建设得到加强。深入推进法治政府建设，政府立法工作富有成效，“六五”普法工作顺利启动，行政复议工作荣获全国先进称号，政府信息公开力度不断加大，人大代表建议和政协提案办理工作全面完成。深入推进服务型政府建设，“发展提升年”活动深入开展，“两集中三到位”执行力度加大，市、县（区）、乡镇（街道）、村（居）四级便民服务体系全面建成。深入推进廉洁政府建设，教育、医疗卫生、公路等领域的突出问题得到有效治理，工程建设、土地领域专项治理深入开展，工程招投标、土地出让、政府采购和产权交易四项制度全面落实。

　　平安创建活动深入开展。“迎城运、保平安”、打击“两抢一盗”、“清网行动”追逃、危险物品管理集中整治、“打四黑除四害”等专项行动成效明显；推进了“天网工程”建设应用，社会治安动态防控体系更加完善。严格落实信访工作“一岗双责”制度，领导包案、积案化解和三级终结工作深入推进。专门成立医患纠纷调处中心，化解医患矛盾。高度重视安全生产，交通、消防、产品质量、食品药品等安全工作得到加强，各类突发事件应急处置能力进一步提升。一年来，全市社会治安和安全生产形势明显好转，社会保持和谐稳定。

　　与此同时，国防建设、国家安全、人民防空、外事侨务、民族宗教、对台事务等工作继续加强。审计、工商、质监、物资、参事、档案、史志、地名、保密、科普、气象、老龄、慈善、妇女儿童、残疾人、社区建设、防灾减灾、军地协作、爱国卫生、红十字会、机关事务、消费者权益保护等工作取得喜人成绩。

　　各位代表，一年来经济社会发展的成绩来之不易，这是省委、省政府和市委正确领导的结果，是市人大依法监督和市政协民主监督的结果，是各民主党派、工商联、无党派人士、各人民团体和驻昌单位、人民解放军、武警官兵以及离退休老同志大力支持的结果，是全市广大干部群众团结一心、奋力拼搏的结果。在此，我谨代表市人民政府，向全市人民，向所有关心、支持南昌发展的同志们、朋友们，表示衷心的感谢并致以崇高的敬意！

　　在看到成绩的同时，我们也清醒地认识到，当前我们面临的最大挑战仍然是发展不足、发展不快，“三个没有根本转变”的格局依然存在，一些深层次矛盾和问题没有得到根本解决。主要表现在：经济总量不够大，综合实力不够强，城市承载功能和管理水平有待提升，资源环境约束压力增强，保障和改善民生任务很重，政务服务和投资环境还要进一步优化。对这些矛盾和问题，我们将采取切实有效措施，认真加以解决。

　　二、2012年工作安排

　　2012年是实施“十二五”规划承上启下的重要一年，也是将南昌打造成为“带动全省发展的核心增长极”的开局之年。

　　展望2012年，世界经济总体形势仍将十分严峻复杂，我国经济发展中不平衡、不协调、不可持续的矛盾和问题仍很突出，经济增长下行和通胀上升的双重压力仍然较大。但总体上看，我国经济长期向好的基本面没有改变，我省加快发展的重要战略机遇期没有改变，我市又好又快发展的总体势头没有改变。具体表现为，党中央、国务院已经定下“稳中求进”的经济社会发展总基调，宏观政策有利；省委、省政府将加大力度支持南昌打造成为“带动全省发展的核心增长极”，发展机遇难得；我市多年积蓄的巨大能量持续释放，内生动力强劲。面对新的形势，我们将保持清醒头脑，积极做好应对各种困难和风险的准备，坚定信心、主动作为，牢固树立“等不起”的发展意识、“慢不得”的危机意识、“坐不住”的责任意识，在宏观调控中抢抓机遇，在区域竞争中加速赶超，不断做大经济总量，提升发展水平，增强竞争实力，矢志不移、竭尽全力为南昌打造成为“带动全省发展的核心增长极”打响“第一枪”，赢得“开门红”！

　　今年政府工作的总体要求是：以中国特色社会主义理论体系为指导，深入贯彻中央经济工作会、省第十三次党代会、市第十次党代会和市委十届二次全会精神，紧紧围绕打造“带动全省发展的核心增长极”的目标，以鄱阳湖生态经济区建设为引领，按照“六个走在全省前列”、“六个率先”和“四个聚焦”的要求，坚持“大投入、大建设、大发展”，着力加强改革创新，着力加快产业发展，着力推进城乡统筹，着力保障改善民生，着力创新社会管理，强力推动经济社会又好又快发展，以优异成绩迎接党的十八大胜利召开。

　　今年经济社会发展的主要预期目标是：

　　地区生产总值增长13%左右；

　　财政总收入增长16%；

　　地方财政一般预算收入增长16%；

　　全社会固定资产投资增长26%；

　　规模以上工业增加值增长18%左右；

　　社会消费品零售总额增长17%；

　　实际利用内资增长12%；

　　实际利用外资增长12%；

　　城镇居民人均可支配收入增长13%；

　　农民年人均纯收入增长13%；

　　万元生产总值能耗下降3.5%；

　　人口自然增长率控制在8‰以内；

　　居民消费价格总水平涨幅控制在4%左右。

　　在具体工作中，我们将把握好以下原则：

　　一是坚持先行先试，创新发展。用足用好用活省委、省政府赋予的先行先试政策，在体制机制、区域合作等方面大胆创新，在产业布局、招商引资等方面率先突破，在破解瓶颈、做强平台等方面敢为人先，在调整结构、自主创新等方面抢先一步，构筑更加开放、更具活力的南昌崛起战略平台。

　　二是坚持产业支撑，提速发展。坚持二产和三产“两业并举”、传统产业和新兴产业“双轮驱动”，大力打造一批“千亿产业集群”、“百亿企业方阵”、“千亿园区板块”、“亿元楼”，实现经济总量迅速扩张、经济结构更加优化。

　　三是坚持要素集聚，集约发展。突出抓好十大工业项目、十大服务业项目、十大城市建设项目、十大城市管理项目、十大社会事业项目、十大民生实事项目，促进资金、技术、信息、人才等要素加速集聚，以大投入带动大建设，以大建设促进大发展。

　　四是坚持改善民生，和谐发展。坚持以人为本、共建共享，更加注重公共服务的均等性，更加注重民生工程的普惠性，更加注重社会管理的创新性，更加注重社会大局的稳定性，不断提高人民群众的幸福感。

　　为实现上述目标任务，我们将重点抓好以下七个方面的工作。

　　（一）突出改革创新，不断提高发展驱动力

　　紧紧抓住省委、省政府支持南昌先行先试、做大做强的重大机遇，最大限度消除发展障碍，最大程度激发发展活力，大力推进改革创新。

　　盘活资金。按照“集中力量办大事，集中财力保重点”的原则，加大财政资金整合力度，扩大扶持企业发展专项资金规模；优化资金使用结构，提高财政资金使用集中度，重点向重大项目倾斜；完善资金使用方式，积极运用贴息、投资补助等政策手段，促进产业投资增长；不断探索行政事业单位资产管理改革，积极整合，逐步注入，支持市级投融资平台做大做强。

　　搞活机制。强力推进国有企业改制重组。加大土地管理体制改革，坚持节约集约利用土地，进一步完善市区两级土地收入分享机制，推广朝阳新城土地储备模式，加大片区土地储备。加快推进户籍制度改革，率先在全省城乡一体化试点改革中取得突破性进展。坚持“非禁即入”，进一步降低门槛，放宽领域，扶持中小企业等实体经济发展，搭建全民创业平台，为非公经济发展创造良好条件。

　　激活平台。做强投融资平台，整合资源，进一步增强融资能力；完善信用担保体系，推进债券发行和上市融资工作，多渠道筹措资金。建立重大项目管理平台，开发产业项目信息动态管理系统，加强对重大招商项目、产业项目、基础设施项目和民生项目的全过程监控。完善土地交易平台，全面实行国有建设用地使用权网上交易。搭建科技创新服务平台，着力为中小企业提供技术成果转化与交易服务、创新创业孵化与融资服务、科技信息搜索与仪器设备共享服务。打造引才用才平台，着力引进一批高层次人才，落实人才培养计划，推动专家服务基地建设。

　　（二）突出产业发展，不断提高发展支撑力

　　产业要发展，关键靠招商，核心是工业，潜力看三产，基础在农业。

　　扩大开放招商。大力实施招商引资“生命线”工程，做好产业招商顶层设计。大力开展产业招商、专题招商、小分队招商、以商招商、商会招商、标准厂房招商，瞄准重点区域、重点企业，力争引进一批投资超10亿、50亿的大项目。加快转变外贸发展方式，鼓励企业开展研发设计、发展自主品牌、大力拓展境外营销。加快建设海铁联运、出口加工区、保税物流中心、南昌国际空港物流城。抓好项目跟踪调度服务，提高项目的履约率、进资率、开工率和投产率。

　　主攻新型工业。着力强化工业核心地位，坚持力量向工业集中、资源向工业配置、资金向工业倾斜，在全市营造“大抓工业、抓大工业”的浓厚氛围。深入实施“双轮驱动”战略，一手抓战略性新兴产业，一手抓传统优势产业。重点发展汽车、航空、光伏光电、软件和服务外包、生物和新医药五大战略性新兴产业，改造提升冶金和新材料、电子信息和绿色家电、机电制造、纺织服装、食品等五大传统优势产业。全力推进南昌航空城、江铃30万辆整车、恒天动力K发动机等十大工业项目建设，着力打造“千亿产业集群”。推动正邦集团、洪都航空等一批重点企业尽快过百亿，着力打造“百亿企业方阵”。坚持突出特色、优势互补、扬优成势，力争每个工业园区重点发展1—2个特色产业，推动园区错位发展，提高工业园区的核心竞争力。

　　提升现代服务业。加快发展商贸物流业。重点打造红谷滩中央商务区，逐步改造提升八一广场中山路市级商业中心以及洪城商圈等6—8个区级商业中心，着力打造总部经济和楼宇经济“亿元楼”，加快推进昌北、昌南、昌西3个物流基地及8个物流中心建设。着力实施“放心粮油六进工程”和农资、日用品、农副产品、再生资源等新农村四大网络建设。着力打造旅游目的地。优化整合旅游资源，完善旅游综合配套，着力打造以滕王阁连接八一起义纪念馆的旅游购物核心街区；加快推进滕王阁扩建、梅岭旅游综合开发、水上游、乡村旅游等项目建设；规划建设以南矶山景区为核心的鄱阳湖生态旅游高地。不断拓展现代金融业。大力推进南昌金融大街建设，吸引银行、证券、保险等金融机构入驻；促进银企合作，争取扩大信贷投放；大力发展创业投资和私募股权投资基金，规范小额贷款公司、担保公司发展；力争2—3家企业上市，支持上市企业再融资。优化提升服务外包业。加快引进服务外包领军企业，大力发展软件外包、呼叫中心、动漫游戏外包等业务模式，积极探索物联网和“云计算”开发应用等相关领域。培育壮大会展经济。精心办好中国绿色食品博览会，积极引进一批大型会展，着力培育1-2个展示南昌、提升形象、促进经济发展的会展节庆品牌。

　　加快发展现代农业。进一步加大农业投入，大力实施农业农村“三大战役”和“十大工程”。稳定粮食、生猪等主要农产品生产，大力发展蔬菜、苗木、花卉、水产等特色农业。做大农业产业化信用担保公司，加强农民专业合作社建设，扶持龙头企业发展。进一步加大农业科技推广力度，加快建设1个国家级、2个省级现代农业示范区。大力实施高标准农田建设，新建扩建14个大型农业综合开发示范项目。大兴农田水利，加快实施中小河流治理、水库除险加固、小农水重点县、农田灌溉“润田”工程等水利建设。

　　（三）突出城市建设，不断提高城市承载力

　　按照“优化规划、拓展规模、强化功能、精细管理、美化环境”的思路，加快提升城市综合承载力。

　　优化规划。围绕“双核三圈五组团”城市空间发展战略，加强中心城区周边地区规划统一管理和市县规划衔接、边界协作，科学编制城市拓展区域控制性详细规划。加快编制城市地下空间利用规划、水环境规划、城市社区规划等各类专项规划。加快修编轨道交通线网规划。

　　拓展规模。按照“多中心、组团式、网络化、生态型”的发展模式，大力拓展城市发展空间。加快新区新城建设。精心打造红谷滩新区，加快开发朝阳洲、红角洲、九龙湖、青山湖西岸，进一步推进五大组团和小城镇建设，让新区和新城成为南昌经济社会发展新的承载空间。加快改造老城区。按照“摸清底数、分类分批、有序推进”的原则，抓紧棚户区和城中村改造，在提升老城品质的基础上，引导老城区的密集人口和优质资源向新城新区转移。

　　强化功能。加快推进沪昆杭南长段、向莆铁路、南昌西客站、火车站东广场、南昌新港枢纽、机场快速通道等重点工程建设，构建高效便捷的现代区域交通运输体系。推进城市方格网干线道路规划建设，加快推进轨道交通、沿江中南大道快速路、城区—县（区）路网畅通工程等“十大城市建设项目”，构建快速通达的城市道路交通体系。加快建设南昌综合客运枢纽、国际集装箱码头。推进城市配套设施同步建设，着力实施新昌电厂二期、燃气一张网、自来水厂、污水处理厂等基础设施建设，加快推进城市干道提升、街区环境整治、城市排水综合整治、垃圾处理、客运场站改造等“十大城市管理项目”，构建配套完善的城市公共服务硬件体系。

　　精细管理。推进城市管理重心下移，适时向城区下放市政道路、园林绿化、排水、城区路灯等城市基础设施的管理和维护权限，进一步理顺城市管理体制机制，实行城管进社区，建立街办、市容、执法三位一体的“三级管理、四级网络”体制。继续推动城市管理社会化、市场化，进一步推进“购买岗位”、“购买服务”、“购买环境”。提升城市管理手段，扩大“数字城管”覆盖面，实行“全时段、全覆盖”监控。按照“差异化、区域化、分类化”的管理标准，严格实行考核、奖惩、问责制，实现城市管理向常态化、长效化、精细化转变。全面开展建筑质量安全大检查，坚决杜绝“豆腐渣”工程。继续深入实施“啄木鸟”行动，持续开展城市环境综合整治，使城市环境保持“永新永亮”。

　　美化环境。深入实施《南昌低碳城市发展规划》，加快推进四大低碳经济示范区建设。深入推进“森林城乡、花园南昌”建设，巩固造林绿化“一大四小”工程成果，着力创建国家生态园林城市和国家森林城市。严守环保底线，加强江河湖泊水系环境综合治理，开展“鄱湖明珠·中国水都”巡活动，让南昌天更蓝、水更清、山更绿、空气更清新、环境更优美。

　　（四）突出城乡统筹，不断提高发展协同力

　　按照“以城带乡、城乡融合”的发展思路，促进城乡资源优化配置，实现城乡统筹协调发展。

　　壮大县域经济实力。按照“应放尽放”和“降低门槛”的原则，进一步扩大县区经济管理权限。加大对县区在财力、融资、电力、路网、基础设施等方面的支持。鼓励城区与县采取“飞地工业”模式，协作共建产业园。支持县区发展“一县一品”和“一县一业”，打造特色产业，力争县区（开发区）主要经济指标增幅达到或超过全省平均水平，在全省县区经济实力排位前移。

　　加快推进村镇建设。大力推动四县城关镇、重点示范镇建设，把小城镇建设与培育特色优势产业结合起来，加强小城镇基础设施和配套功能建设，突出差异化发展，着力打造一批主业突出、各具特色的工业重镇、商贸强镇、生态美镇、旅游名镇、物流活镇。继续实施农村清洁工程，推进农村垃圾无害化处理。推进650个新农村村点建设，打造一批集村庄规建、特色产业、休闲农业、生态优化、乡风文明“五位一体”的综合示范村。

　　（五）突出民生普惠，不断提高公共服务力

　　按照“广泛征询、按需实施，量力而行、尽力而为”的原则，更加注重保障改善民生，更加注重社会建设，努力解决人民群众最关心、最直接、最现实的利益问题。

　　大力推进民生工程。把新增财力更多的投向民生领域，全力实施涉及人民群众切身利益的十大民生实事和政策性民生项目。重点是，就业创业方面：新增城镇就业6.99万人,新增转移农村劳动力3.33万人, 零就业家庭就业安置率100%。新增小额贷款2.5亿元。社会保障方面：实现对农村适龄居民和城镇居民养老保险全覆盖，提高企业退休人员基本养老金水平。社会救助方面：全面启动城市居民家庭经济状况核对工作，提高低保标准，落实优抚政策。加强法律援助。执行计划生育奖励制度。教育助学方面：继续落实义务教育“两免一补”政策。做好贫困家庭学生高考入学政府资助等济困助学工作。组织实施农村义务教育学生营养餐试点工作。公共卫生方面：提高城镇居民基本医疗保险补助标准和新型农村合作医疗补助标准，资助困难企业职工参加城镇职工医疗保险，提高医疗保险统筹层次；组织实施好“光明·微笑”行动、儿童“两病”免费救治、尿毒症患者免费透析救治、重度聋儿人工耳蜗植入和康复救治工作。住房保障方面：扎实推进保障性住房建设，确保完成省下达任务，做好经济适用住房补贴和廉租住房租赁补贴的发放工作。文化体育方面：建设农家书屋、社区书屋，巩固县区文化馆、博物馆、图书馆和乡镇（街办）文化站建设改造成果，开展送戏下乡、放映电影活动。实施全民健身路径工程。群众生产生活方面：提高企业职工最低工资标准。整体推进100个扶贫重点村的扶贫开发。抓好粮油等生活必需品物资供应，完善价格应急监测预警体系，切实把物价总水平稳定在可控范围之内。

　　全面发展社会事业。坚持科教兴市。出台并实施中小学网点规划，加速拓展优质高中规模，积极推进义务教育均衡化。大力实施学前教育发展三年行动计划，新建、改扩建一批公办幼儿园，启动中小学生社会实践基地建设，大力实施教育“五个十”工程。加快建设国家创新型试点城市，大力建设3个国家级研发平台，加快组建30个技术创新团队和6个知识创新团队，培育10家以上省级创新型试点企业。推进南昌科技创新服务中心建设。组织实施科技产业化重大项目，继续实施“十城千辆”、“十城万盏”工程。推进文化强市。大力发展文化产业，制定文化产业发展规划，建立文化产业分类指标体系和文化产业绩效考核体系，做强一批龙头文化企业，打造红谷创意、泰豪动漫、八大山人、文港文化用品等文化产业园；推出一批文化品牌，重点打造南昌采茶戏、南昌清音、南昌瓷板画等；加强文化设施建设，加快推进彭友善美术馆、市群众艺术馆新馆、市博物馆新馆等建设，推进文化惠民工程，促进文化事业繁荣发展。建设健康城市。加快推进市三医院朝阳院区、市洪都中医院新院、江西省皮肤病专科医院综合大楼、市第一医院东院等项目建设；着力落实国家基本药物制度，创建全国食品药品安全示范区，创建国家、省级慢性非传染性疾病综合防治示范区。精心办好第15届南昌市运动会；着力打造国家全民健身示范城市。严格人口与计生工作目标管理，坚决落实“一票否决制”。大力开展爱国卫生运动。进一步加强国防教育和“双拥”工作，争创“全国双拥模范城市”七连冠。

　　（六）突出社会管理，不断提高社会凝聚力

　　坚持把创新社会管理作为统筹经济社会发展的重要举措和维护社会稳定的根本要求，扎实推进，真正实现服务民众、促进民和、维护民利、保障民安。

　　加强基层建设。全面完成村（居）委会换届工作，积极稳妥做好市域内行政区划调整等工作；全面落实改制后的国企职工及生活区社会化管理；发展“村（居）民议事会”等村（居）民自治的新社会组织，支持引导中介、协会等社会组织健康发展，促进民族和睦、宗教和顺、社会和谐。

　　加强综合治理。深化平安南昌创建，继续加强普法工作。深化社会矛盾化解、社会管理创新、公正廉洁执法三项工作，推进公安信息化、执法规范化建设，开展“三访三评”，深化“大走访”活动；加强流动人口服务和管理，加强对刑释解教和社区矫治等特殊人员的帮教管理。深入开展“打黑除恶”、打击“两抢一盗”、禁毒禁黄等专项斗争，打击拦路堵路等不法行为。

　　加强信访维稳。坚持用群众工作统揽信访工作，畅通信访渠道，引导群众理性合法表达利益诉求，积极预防和妥善处置群体性事件；健全第三方参与接访、信访事项督查督办、“三级终结”等机制，坚持领导信访接待日、重大信访事项领导包案、“一岗双责”等制度，深化矛盾纠纷排查，加强医患纠纷等调处工作，努力把矛盾解决在基层、化解在萌芽状态。

　　加强安全工作。全面落实安全工作辖区负责制、主管部门负责制、行政首长负责制，毫不松懈地抓好生产安全、消防安全、交通安全、食品药品安全、产品质量安全。深入开展隐患排查，实行重大隐患“挂牌督办”，坚决遏制各类重特大安全事故发生。切实加强应急管理，不断提高应急救援和应急处置能力。

　　（七）突出效能提升，不断提高政府公信力

　　坚持把效能提升作为提高政府公信力的突破口，做到让组织满意、群众满意、基层满意、客商满意，努力打造“法治、务实、高效、廉洁”政府。

　　坚持科学理政。坚持科学决策、民主决策、依法决策，把公众参与、专家论证、风险评估、合法性审查和集体讨论决定作为重大决策的必经程序，增强各项工作决策的科学性、预见性和可操作性。

　　坚持依法行政。加强政府立法，严格规范性文件管理，落实行政执法责任制，促进行政行为规范有序。主动接受市人大的法律监督、工作监督和市政协的民主监督，认真办理人大代表建议和政协提案，广泛听取各民主党派、工商联、无党派人士、群众团体和社会各界的意见建议，自觉接受新闻媒体和社会公众的监督。大力加强诚信建设，深入推进政务公开，切实保障公众知情权、参与权和监督权。

　　坚持高效勤政。进一步加强机关效能和干部作风建设，在全市范围内开展“作风大转变、效能大提速、环境大优化”集中整治活动。进一步精简会议和文电，简化公务接待，集中精力察实情、出实招、办实事、求实效。进一步树立“五星级”服务理念，认真为企业和基层排忧解难，切实保护投资者的合法权益。强化行政问责制度，对损害投资环境的人和事，发现一起，曝光一起，查处一起，真正做到亲商、安商、富商。进一步推进行政审批制度改革，精简行政审批事项和非行政审批事项，优化工作流程，开展电子政务，提高行政效能。

　　坚持廉洁从政。严格执行党风廉政建设各项规定，全面落实党风廉政建设责任制，深入推进风险岗位廉能管理工作。坚持勤俭节约，降低行政成本。强化行政监察和审计监督，加大重点领域、关键环节专项治理力度，严肃查办违纪违法案件，营造风清气正的政务环境。

　　各位代表，站在新的历史起点，面对省委、省政府的新要求和全市人民的新期待，把南昌建设成为“带动全省发展的核心增长极”，我们深感责任重大、任务艰巨、使命光荣。让我们更加紧密地团结在以胡锦涛同志为总书记的党中央周围，在省委、省政府和市委的坚强领导下，抢抓机遇，团结拼搏，开拓创新，务实奋进，为推动南昌经济社会又好又快发展，加速科学发展、进位赶超、绿色崛起的进程而不懈努力，以优异的成绩迎接党的十八大胜利召开!
